# The Interplay between Immune System and Microbiota in Osteoporosis

**DOI:** 10.1155/2020/3686749

**Published:** 2020-02-26

**Authors:** Pietro Locantore, Valeria Del Gatto, Silvia Gelli, Rosa Maria Paragliola, Alfredo Pontecorvi

**Affiliations:** Fondazione Policlinico Universitario Agostino Gemelli–IRCCS, Università Cattolica del Sacro Cuore, Rome, Italy

## Abstract

Osteoporosis is a disease characterized by low bone mass and alterations of bone microarchitecture, with an increased risk of fractures. It is a multifactorial disorder that is more frequent in postmenopausal women but can be associated to other diseases (inflammatory and metabolic diseases). At present, several options are available to treat osteoporosis trying to block bone reabsorption and reduce the risk of fracture. Anyway, these drugs have safety and tolerance problems in long-term treatment. Recently, gut microbiota has been highlighted to have strong influence on bone metabolism, becoming a potential new target to modify bone mineral density. Such evidences are mainly based on mouse models, showing an involvement in modulating the interaction between the immune system and bone cells. Germ-free mice represent a basic model to understand the interaction between microbiota, immune system, and bone cells, even though data are controversial. Anyway, such models have unequivocally demonstrated a connection between such systems, even if the mechanism is unclear. Gut microbiota is a complex system that influences calcium and vitamin D absorption and modulates gut permeability, hormonal secretion, and immune response. A key role is played by the T helper 17 lymphocytes, TNF, interleukin 17, and RANK ligand system. Other important pathways include NOD1, NOD2, and Toll-like receptor 5. Prebiotics and probiotics are a wide range of substances and germs that can influence and modify microbiota. Several studies demonstrated actions by different prebiotics and probiotics in different animals, differing according to sex, age, and hormonal status. Data on the effects on humans are poor and controversial. Gut microbiota manipulation appears a possible strategy to prevent and treat osteopenia and/or osteoporosis as well as other possible bone alterations, even though further clinical studies are necessary to identify correct procedures in humans.

## 1. Introduction

Osteoporosis is the most common bone disorder characterized by low bone mass and microarchitectural deterioration of bone tissue, with increased risk of fractures.

Fractures severely affect patients' quality of life and mortality, especially in case of major fractures (femur and vertebrae) and represent a serious public health problem due to population aging, with high impact on the health care costs. In fact, the incidence of osteoporotic fractures is rapidly increasing in both sexes because of longer life expectancy [1].

Osteoporosis is classically distinguished in primary and secondary. Primary form includes postmenopausal osteoporosis, due to the fall of estrogen levels. Secondary form is due to endocrine diseases (i.e., hypercortisolism, hyperthyroidism), kidney diseases, hematologic diseases (i.e., multiple myeloma and malignant neoplasms infiltrating the bone), autoimmune or rheumatic diseases (i.e., inflammatory bowel disease, rheumatoid arthritis), drugs (i.e., steroids), malnutrition, malabsorption (i.e., celiac disease), or prolonged immobilization [2].

Bone loss is an asymptomatic process, so that the diagnosis of osteoporosis may often be made only after a fracture has occurred. Fractures can be prevented by reducing the risk of falling, changing lifestyle and nutrition, smoking, and alcohol abstention [3]. In case of vitamin D deficiency, osteoporosis is more frequent. Therefore, the first line treatment is characterized by calcium and vitamin D supplementation, which is essential for a good bone activity [4]. Calcium may be taken with food and tablet. In addition, several drugs are available to treat osteoporosis and reduce the risk of fracture blocking bone reabsorption (such as bisphosphonates and denosumab), by stimulating bone formation or both (such as teriparatide or abaloparatide).

However, such drugs have safety and tolerance problems in long-term treatment. Concerns about rare side effects of antiresorptive drugs (osteonecrosis of the jaw, gastritis, and atypical fractures) lead many patients to discontinue such therapy [5]. Therefore, new tools are necessary to develop new treatments. These new options should have low side effects, improved efficacy, and adherence to treatment as well as overall patient outcome.

Recently, gut microbiota (GM) has been highlighted to have strong influence on bone metabolism, attracting the attention of endocrinologist and gastroenterologist as a potential new target to modify bone mineral density. The basis of these evidences are mainly focused on an involvement in modulating the interaction between immune system and bone cells [6–9].

GM is composed by all commensal, symbiont, and pathogenic microorganism consisting of bacteria, fungi, and viruses that colonize human intestine. GM is acquired at birth, mainly from the mother, and it is influenced by several factors such as genetic background, diet, age, eventual treatments, and antibiotics [10–12]. GM differs among people, and it is important to have a coexistence of different phyla in the intestine. Scientific community is increasing interest in studying such new “organ” to deeply understand its role and potentiality to treat diseases. It is strongly involved in human development, especially of the immune system; in fact, GM is necessary for an appropriate education and evolution of the innate and adaptive immune response [13].

GM plays an important role in maintaining gut barrier function, protecting the host against pathogens, food digestion, and modulating systemic immune responses by interacting with dendritic cells, macrophages, granulocytes, T- and B-cells, and intestinal epithelial cells [13].

The relationship between host and GM is complex and is based on variety of interactions, which are mainly controlled by the immune system. In case of alteration of the GM, such homeostatic balance may be interrupted, and the host may develop some pathologic conditions. A lack of variety among germs is a risk factor for the development of diseases (mainly immune mediated disorders) such as obesity [14, 15], insulin resistance [16, 17], inflammatory bowel diseases [18, 19], neurodegenerative disorders [20], and other metabolic diseases [21].

The intent of this review is to expose the mechanism underlying the interaction between GM and osteoporosis.

## 2. Studies on Germ-Free Models

The role of GM has been investigated looking at germ-free mouse models. These mice are raised in sterile cages, so that they cannot acquire any germ in the gut. They grow up weak, with a deficient formation of immune system and lymphoid organs. In this model, data on bone density are controversial, as in some studies, germ-free mice showed a low bone mass, while in other papers, they presented an increased bone mass density compared to normal mice.

Schwarzer et al. [22] observed that male germ-free mice presented a very weak bone development, including femur length, cortical thickness, and cortical/trabecular bone fraction. This condition has been supposed to be linked to low IGF1 levels that have been documented in such models [23].

Instead, Sjögren et al. [24] demonstrated that female germ-free mice presented an increased bone mineral density and a lower number of osteoclasts compared with conventionally raised mice. Moreover, these models are protected from developing osteoporosis in steroid deprivation settings. In fact, ovariectomy does not induce bone loss on germ-free mice [25].

The same controversial data have been reported in mice treated with oral antibiotics, in which GM is severely affected [26]. Several unclear pathways have been found in these opposite results; for that, such differences may be due to the lack of standardization among studies, differing for mouse breed, age, sex, antibiotics used, and technic of checking bone mineral density.

Male mice treated with antibiotics presented a decreased bone density, while female mice had an increased bone density [27]. On the basis of this, it is possible that GM composition and antibiotics response may be influenced by sexual hormones or other sex-related factors.

Anyway, Yan et al. [23] demonstrated that subsequent colonization of germ-free mice, with a normal GM composition, caused a reduction of bone mass in short period. In fact, analysis of bone density one month after colonization showed a reduction of bone mass, while 8 months after colonization, mice showed a bone density which was comparable with mice raised in conventional condition.

## 3. Role of Gut Microbiota in Vitamin D and Calcium Absorption

Vitamin D sufficiency and normal calcium phosphate metabolism play a key role in developing and holding an appropriate bone mass. A correct absorption of such trace elements is crucial.

The active form of vitamin D is 1,25-dihydroxyvitamin D (1,25(OH)2D3). It is produced in the skin after sun exposition or absorbed from diet and activates the vitamin D receptor (VDR). VDR is a nuclear receptor and transcription factor expressed in a wide variety of tissues, including the intestine, and it modulates metabolic and immune system processes.

Classically, vitamin D is known to regulate bone development and calcium homeostasis through its actions in the intestine, kidney, and bone. Vitamin D regulates calcium absorption in the intestine and kidney by activating transcellular calcium channel (TRVPV and CalbindingD9K) which mediates intracellular calcium diffusion [28].

Low levels of vitamin D or inactivating polymorphisms in VDR have been associated with inflammatory and metabolic disorders. In particular, the variation of human VDR gene shapes the gut microbiome at the genetic level. In fact, it has been observed that mouse models carrying VDR deletion in gut epithelia cells present dysbiosis and an increased susceptibility to inflammatory bowel diseases [29]. Furthermore, vitamin D induces the expression of cathelicidin antimicrobial peptide (CAMP) gene, which is expressed by immune and epithelial cells and enhances barrier function, suggesting that vitamin D has antibacterial effects [30]. Moreover, vitamin D reduces the permeability of intestinal cells in animal models of colitis [31].

However, data on biological function of vitamin D and VDR in GM are limited. The GM has effects on the host immune system as well as vitamin D, and it also plays a critical role in the synthesis of vitamins and trace elements. For that, there is a strong direct interaction between vitamin D levels and GM composition, also influencing calcium absorption. A reduction in the production of 1,25(OH)2D3 may lead to gut inflammation and a shift in the balance of the GM composition [32].

## 4. Role of Gut Microbiota in Bone Homeostasis

Bone is a dynamic tissue whose homeostasis is based on several different mechanisms. A wide variety of factors influences bone strength such as hormones, physical activity, diet, weight, and lifestyle. Moreover, bone metabolism seems to be influenced by several gastrointestinal peptides, such as ghrelin, peptide tyrosine-tyrosine (peptide YY), incretins, glucose-dependent insulinotropic polypeptide or gastric inhibitor polypeptide (GIP), and glucagon-like peptide (GLP) 1 and 2 [33].

The main factor involved on bone density is the balance between the osteoblastic (that replace bone) and osteoclastic (that reabsorb bone) activity. This process, called remodelling, is necessary to build the skeleton during growth, regulate calcium homeostasis, and repair microdamages. Remodelling involves multiple molecular events, such as cooperation between osteoblasts, osteoclasts and other cell populations (i.e., immune cells), and several hormones such as parathyroid hormone (PTH), vitamin D, calcitonin, growth hormone (GH and IGF1), sexual hormones, growth factors and cytokines. An increased osteoclast activity or reduced osteoblast activity can cause reduction in the architecture of bone mass, causing osteoporosis. In this setting, risk of fracture is increased.

Remodelling is triggered by signalling from osteoblasts and osteocytes that produce receptor activator of nuclear factor kB ligand (RANKL), a member of the tumor necrosis factor (TNF) receptor family, which binds to the RANK receptor on osteoclast precursors. This binding is essential for activation of osteoclast precursors to mature form which can destroy bone matrix.

Osteoclasts attach firmly to the bone surface and secrete hydrochloric acid and cathepsin K to dissolve bone mineral. After resorption is complete, osteoblasts lay down bone collagen matrix, which is then mineralized.

GM has supposed to influence bone homeostasis through the effect on the systemic immunity. In fact, microbiota is involved in production of circulating cytokines and development of lymphoid cells, particularly of T helper 17 (Th17) lymphocytes. This correlation between GM and immune system is important, because the latter plays an essential role in regulating bone density. In fact, RANKL is expressed not only by mesenchymal cells, osteoblasts and osteocytes, but also by activated T CD4+ lymphocytes, indicating that this is a molecule that bridges the skeletal and immune systems [34]. Moreover, lymphocytes produce tumor necrosis factor *α* (TNF*α*) and interleukin (IL)-17, both involved in osteoclastogenesis [35, 36]. However, T-cells also produce interferon gamma (IFN-*γ*), which counterbalances the action of RANKL, determining an inhibitory effect on osteoclastogenesis. The only osteoclastogenic Th subset is represented by Th17 cells through the production of IL-17 that induces the expression of RANKL. Th17 cells are a subset of proinflammatory T helper cells which play an important role in maintaining mucosal barrier and preventing intestinal colonization by pathogenic germs. They have also been implicated in autoimmune and inflammatory disorders. Th17 cells activated by intestinal inflammation migrate into the bone matrix, where IL-17 enhances local inflammation leading to an increase of inflammatory cytokines, such as TNF*α* and IL-1, raising RANKL expression and activating osteoclast precursor cells [37]. In vivo, Th17 have been associated to increased osteoclast differentiation both in mouse models and in humans affected by inflammatory diseases. In fact, Th17 lymphocytes were also detected in peripheral blood of patients suffering from Crohn's disease, and for that, Th17 may be involved in decreased bone density frequently detected in these patients [38]. Th17 lymphocytes may be a promising therapeutic target for the bone reabsorption associated with T-cell activation. It is also remarkable to point out that germ-free mice do not present Th17 cells in their bowel tissue, but their development and maturation may be induced by germ colonization.

Moreover, the relationship between GM and bone is also mediated by innate immunity through several receptors such as nucleotide-binding oligomerization domain proteins (NOD1 and NOD2) receptors and Toll-like receptor 5 (TLR5). NOD1 and NOD2 are ubiquitary intracellular sensors of pathogen-associated molecular patterns (PAMPs), mainly expressed on epithelial and immune cells, that bind bacterial peptidoglycans and activate the NFkB pathway playing a key role in the effects of microbiota on bone. In fact, neither the expression of TNF*α* and RANKL nor the bone density is affected by the microbiota variations in mice which are knocked out for these two genes [39].

TLR5 is the innate immune receptor known to recognize flagellin, one of the main bacterial proteins. It is expressed on both immune and not-immune cells, such as enterocytes. A recent study has identified TLR5 as a new mediator in the process of inflammation-induced bone loss and osteoclastogenesis, through the activation of RANKL pathway [40]. Mice that are knocked out for this receptor develop deficiencies in the immune system leading to change in the GM composition, with the prevalence of *Proteobacteria*, *Firmicutes*, and flagellated bacteria that invade bowel mucosal barrier [41]. Therefore, these mice exhibit hyperphagia, obesity, insulin resistance, hypertension, hyperlipidemia, and increased inflammation, due to GM imbalance and show a decreased bone strength, while mice lacking TLR5 that are raised in germ-free condition do not present the metabolic phenotype [42]. The use of antibiotics leads to a greater reduction of the whole-bone femur bending strength in mice knocked out for this receptor with respect to wild type [26].

Another important molecule implicated in immunomodulation is Lipopolysaccharides (LPS), which is the main component of bacterial cell wall in gram-negative bacteria. Such molecule stimulates inflammation by activating transformed growth factor (TGF) and Toll-like receptors 4 [43]. LPS has been documented to be involved in bone metabolism. A mouse model, in fact, was implanted with LPS to induce inflammation [44]. These mice showed femoral bone loss, suggesting a potential role of LPS in reducing bone mineral density. In mice treated with high dose LPS, trabecular bone volume of the proximal tibial metaphysis tended to be decreased, while an upregulation of the inflammatory mediators, interleukin-1, cyclooxygenase-2, and TNF was found.

Anyway, the major cause of osteoporosis is sexual hormone deficiency, mainly estrogen lack in postmenopausal women. During menopause, there is a progressive bone loss, due to the upregulation of osteoclast maturation and activity mediated by several cytokines. In fact, estrogen deficiency leads to an alteration of the immune response and enhances the production of TNF*α*, which directly induces osteoclast differentiation and indirectly increases the expression of RANKL and macrophage colony-stimulating factor (M-CSF) by monocytes and T-cells. Moreover, estrogen deficiency increases bowel permeability and promote inflammation. Furthermore, the lack of estrogens increases the expression of Class II TransActivator (CIITA), a transcriptional factor involved in the upregulation of major histocompatibility complex class II on macrophages, improving the antigen presentation [45]. Mouse models of osteoporosis caused by ovariectomy suggest that osteoclast activity is mainly stimulated by activated T-cells, which promote macrophage differentiation and the expression of M-CSF and RANKL by stromal cells, through the activation of the CD40/CD40L system [46]. Ovariectomy increases T-cell activation through the upregulation of several intracellular pathways, such as STAT3, ROR-ct, and ROR-a, and the downregulation of Foxp3 [47]. Several studies suggest that T CD4+ lymphocytes are the most relevant source of TNF*α* in condition of estrogen deficiency. Moreover, the lack of T-cell in nude mice seems to preserve against postovariectomy bone loss. However, the transfer of wild-type T-cells restores the capacity of ovariectomy to induce bone loss. Such alteration of the immune system has also been demonstrated in humans and seems to be more relevant in osteoporotic women [48]. Furthermore, in postmenopausal women, hormone replacement therapy decreases the production of osteoclastogenic cytokines [49].

In this contest, GM is central in controlling lymphocytic activation on the basis of sexual hormonal change. The same data have been confirmed in mouse models treated with leuprolide and gonadotropin realising hormone agonist inducing menopause. Moreover, a pilot study [50] conducted on a small number of patients has shown GM modification among osteopenic patients, osteoporotic patients, and control. Such preliminary data suggest a connection between GM and osteoporosis, but further investigations are needed to confirm this hypothesis. However, in our opinion, it is reasonable to speculate that dysbiosis may exacerbate the bone loss in postmenopausal women.

Recent studies have confirmed a close connection between GM and bone diseases. This linkage is not only limited to disorders connected to hormonal changes but may include a huge number of different pathways all associated by inflammation. In fact, GM modification has been linked to several rheumatic and autoimmune diseases, and the inflammation is one of the factors that contributes to osteoporosis in patients with inflammatory bowel diseases. In fact, cytokines produced during intestinal inflammation may alter osteoblast action and bone density. Furthermore, it has been observed that osteoporotic patients with inflammatory bowel diseases have higher circulating proinflammatory cytokines levels. In this contest, TNF antagonists (infliximab and adalimumab), used as conventional treatment for inflammatory bowel diseases, appear to have beneficial effects on bone metabolism, increasing bone formation. It has also been observed that TNF blockade leads to an important increase of bone formation markers, such as osteocalcin and procollagen type 1 N-terminal propeptide, and to a stabilization of bone mass density [51]. Moreover, TNF inhibitors seem to influence the GM composition. Indeed, mice treated with TNF inhibitors have shown alterations of the GM, with differences between genders and age. Thus, you can speculate that the effect of TNF blockade on bone may also be mediated by the modulation of GM. However, available data are still limited.

Another important model to support the connection between GM and bone is characterized by patients affected by small intestinal bacterial overgrowth syndrome. This syndrome is characterized by malabsorption due to destruction of nutrients by bacteria, causing alteration in intestinal calcium and vitamin D absorption. These patients present a chronic bowel inflammatory status and develop bone alteration such as osteomalacia.

## 5. Prebiotics

Prebiotics are fermentable food ingredients that cannot be digested by humans while stimulate the growth and activity of GM as substrate of their metabolism. After this process, GM produces specific metabolic products that can be subsequently used by the host. Prebiotics include a large group of nondigestible oligosaccharides composed by short-chain sugar, the most common of which are galactooligosaccharides (GOS), fructooligosaccharides (FOS), inuline, xylooligosaccharides (XOS), polydextrose, and lactulose. In the group of prebiotics, a list of metabolizable food ingredients can also be included, as compounds of human milk, onions, garlic, and other vegetables.

Prebiotics are safe and can be given to any age after the fifth month of life. The only side effect may be bloating, gas, and increased bowel movements.

Fermentation of fibres in the large intestine causes the production of short-chain fatty acid (SCFA), such as acetate, propionate, valerate, isovalerate, butyrate, and isobutyrate [52]. These molecules increase calcium intestinal absorption reducing bowel pH and promote the gut villi development, causing GM modifications. They also improve the deconjugation of phytoestrogens and may modulate immune system [53].

In female mice, the use of prebiotics reduces bone loss due to estrogens deficiency after ovariectomy [54]. Estrogens lack, in fact, causes reduction in calcium absorption. The administration of inuline and FOS have been demonstrated to increase calcium absorption in ovariectomized rats [55].

Administration of GOS to male rats induces a pH decrease in gross intestine, an increase of bifidobacteria, and calcium and magnesium absorption leading to improvement of bone density [56]. Anyway, it is not clear if such increased calcium absorption also causes an increase in BMD in all animals (such effect has been demonstrated in mice and rats but not in pigs).

In humans, the effect of FOS administration is controversial. In fact, administration of one-year treatment did not modify bone density in adolescent girls [57], while it reduces bone loss in postmenopausal women [58].

In male mice, ingestion of FOS increases cortical and trabecular bone [59]. Ingestion of food with a high amount of fibres improves cortical thickness, cortical bone mineral content, bone strength, and trabecular BMD in rats. The same evidence has been found in case of GOS supplementation [56].

Lactitol is a nonabsorbed sugar that can increase calcium absorption in rats reducing intestinal pH, which raises calcium bioavailability [60].

Milk of mothers with new born affected by malnutrition are poorer of human milk sialylated oligosaccharides, an important energy source for gut bacteria, and this condition may affect bone health. In fact, germ-free mice colonized with stools from malnourished Malawian infants showed a delayed growth that improved after administration of sialylated milk oligosaccharides [61].

Effects of prebiotics on bone homeostasis are controversial and seem to be related among others to the type of prebiotic. A recent study has been demonstrated that administration of agave fructans and inulin increases serum osteocalcin levels in female mice [55], while a GOS/FOS and calcium combination increased bone mineralization [62]. In contrast, the use of inulin and FOS documented an increased bone resorption in ovariectomized rats [63]. In postmenopausal women treated with FOS for 2 years, a decrease of serum and urine bone turnover markers was recorded [58]. Another important tool to improve bone density is represented by the use of FOS in combination with soy isoflavone treatment in ovariectomized rats, which has shown to decrease bone resorption improving BMD [64].

Prebiotics can also alter the GM composition. In fact, FOS and GOS increase the proportion of bifidobacteria in GM, affecting the rate of production of SCFA [52]. The direct connection between prebiotics and bone is not fully clear, but it is definitively a promising research field due to the effect on local GM and its metabolites.

## 6. Probiotics

Probiotics are live microorganism that, if administrated in appropriate amount, can provide health benefits to the host. Several species of microbes can be defined as probiotics, such as *Lactobacilli*, *Bifidobacteria*, *Escherichia*, *Enterococcus* and *Bacillus subtilis*, and *Saccharomyces*.

Probiotics are usually provided in dairy products, such as yoghurt as concentrated cultures or as inoculants in milk-based food or dietary supplements in form of capsules, bags, or tablets. Recently, they have been added to new products, such as ice cream, beer, and toothpaste.

Their effect on bone status has been extensively studied, both in mice and in human. In particular, *Lactobacillus reuteri* was able to improve bone mineral density in ovariectomized murine models, even in the absence of milk [65], but it does not have effects on female intact mice [66]. However, it is interesting to note that *Lactobacillus reuteri* has been shown to increase bone mineral content in male mice [66], confirming the role of sex hormones in the probiotics effect on bone. Moreover, this bacterium has been supposed to have an anti-TNF*α* activity, modulating bone metabolism through the immune system [67].

Similar effects have been observed in case of oral supplementation of *Lactobacillus paracasei* and *helveticus* that affect osteoclastogenesis decreasing the production of IL1*β* and TNF*α* in ovariectomized murine models [68].

Another study on sex hormone-deficient mice has demonstrated that twice-weekly treatment with *Lactobacillus rhamnosus* inhibits bowel inflammation and decreases bone loss through a reduced expression of RANKL and TNF*α*, unlike what happens using of nonprobiotic breed of *Escherichia coli*. Moreover, this study has confirmed that estrogen deficiency affects gut barrier integrity leading to immune system activation. In fact, hypogonadal germ-free mice did not exhibit the same bone damage of wild-type hypogonadal mice [69].

Decreased bone loss due to sex-steroid lack seems to be partially restrained also with the use of *Bifidobacterium longum* in ovariectomized rats [70].

As to the effect on humans, it has been recently demonstrated that the oral administration of *Lactobacillus reuteri* in postmenopausal women increases tibial bone density [71] and circulating vitamin D levels [72]. *Lactobacillus rhamnosus* and *Lactobacillus plantarum* also increase serum vitamin D, through a higher expression of VDR in both mouse and human enterocytes [73]. VDR knockout animals exhibit a decreased presence of lactobacilli compared to clostridium and bacteroides [74]. Furthermore, these models do not benefit of the protective effect of probiotics on Salmonella infections.

*Bifidobacteria* have also demonstrated healthy effects in yoghurt consumers. However, not all dairy products have the same effect on bone metabolism. In fact, the Framingham Offspring Study has highlighted that yoghurt and milk absorption improves hip but not spine bone density [75]. The positive effect of yoghurt was also confirmed in elderly people. Indeed, high yoghurt intake led to better physical performances and higher bone mineral density [76].

Furthermore, the use of probiotics may be important also in oral pathologies. In fact, in rats affected by periodontitis, oral *Saccharomyces cerevisiae* administration as monotherapy or in combination with standard therapy improves local inflammation and decreases alveolar bone loss [77].

It has also been suggested that combined use of probiotics and prebiotics, called symbiotics, may increase effects on bone homeostasis. For that, Michaëlsson et al. [78] have studied the effect of high intake of fruits and vegetables in combination with fermented dairy products (i.e., yoghurt) in postmenopausal women. They observed that high absorption of symbiotics reduces the risk of hip fracture more than low intake of vegetables, fruit, and fermented milk. However, it is important to note that the beneficial effect of prebiotics is considerably increased by concomitant assumption of probiotics, whereas the use of probiotics alone has already a notable effect on bone mineral content.

## 7. Conclusions

Osteoporosis is a multifactorial disorder associated with reduced bone density and high risk of fracture. Such condition is more frequent in postmenopausal woman but can be associated to other disease (inflammatory bowel disease, celiac disease, etc.).

Several studies have defined a central role of GM in the modulations of immune response in regulating bone activity, mainly in mouse models. GM manipulation appears to be a possible strategy to prevent and treat osteopenia and/or osteoporosis as well as other possible bone alterations, even though further clinical studies are necessary to identify correct procedures in humans. GM modification may play a role together with diet, lifestyle, and drugs.

The chance of using appropriate prebiotics and probiotics to increase bone density in different ages is also a possible new path that may be followed in the next few years, and the role of dairy products is still central. Another possible option is the development of functional food to improve prebiotic effects.

GM transplant is another option that may be considered in severe diseases. At present, its role is clear in treating antibiotic resistant colitis infections, but the chance of using GM transplant for treating bone disease needs further investigations.
